# Inhibition of *PHLDA3* expression in human superoxide dismutase 1-mutant amyotrophic lateral sclerosis astrocytes protects against neurotoxicity

**DOI:** 10.1093/braincomms/fcae244

**Published:** 2024-07-25

**Authors:** Kornélia Szebényi, Ingrid Vargová, Veselina Petrova, Jana Turečková, George M Gibbons, Monika Řehořová, Mai Abdelgawad, Alexandra Sándor, Dana Marekova, Jessica C F Kwok, Pavla Jendelová, James W Fawcett, András Lakatos

**Affiliations:** Department of Clinical Neurosciences, John van Geest Centre for Brain Repair, University of Cambridge, Cambridge, CB2 0PY, UK; Research Centre of Natural Sciences, Institute of Molecular Life Sciences, Budapest, 1117, Hungary; Institute of Experimental Medicine, Czech Academy of Sciences, Prague, 142 20, Czech Republic; Department of Clinical Neurosciences, John van Geest Centre for Brain Repair, University of Cambridge, Cambridge, CB2 0PY, UK; Institute of Experimental Medicine, Czech Academy of Sciences, Prague, 142 20, Czech Republic; Department of Clinical Neurosciences, John van Geest Centre for Brain Repair, University of Cambridge, Cambridge, CB2 0PY, UK; Institute of Experimental Medicine, Czech Academy of Sciences, Prague, 142 20, Czech Republic; Second Faculty of Medicine, Charles University, Prague, 150 06, Czech Republic; Department of Clinical Neurosciences, John van Geest Centre for Brain Repair, University of Cambridge, Cambridge, CB2 0PY, UK; Research Centre of Natural Sciences, Institute of Molecular Life Sciences, Budapest, 1117, Hungary; Doctoral School of Molecular Medicine, Semmelweis University, Budapest, 1085, Hungary; Institute of Experimental Medicine, Czech Academy of Sciences, Prague, 142 20, Czech Republic; Institute of Experimental Medicine, Czech Academy of Sciences, Prague, 142 20, Czech Republic; School of Biological Sciences, University of Leeds, Leeds, LS2 9JT, UK; Institute of Experimental Medicine, Czech Academy of Sciences, Prague, 142 20, Czech Republic; Second Faculty of Medicine, Charles University, Prague, 150 06, Czech Republic; Department of Clinical Neurosciences, John van Geest Centre for Brain Repair, University of Cambridge, Cambridge, CB2 0PY, UK; Institute of Experimental Medicine, Czech Academy of Sciences, Prague, 142 20, Czech Republic; Department of Clinical Neurosciences, John van Geest Centre for Brain Repair, University of Cambridge, Cambridge, CB2 0PY, UK; MRC-WT Cambridge Stem Cell Institute, Biomedical Campus, Cambridge, CB2 0AW, UK

**Keywords:** astrocyte, amyotrophic lateral sclerosis, PHLDA3, astrocyte–neuron interaction, cell stress

## Abstract

Pleckstrin homology-like domain family A—member 3 (PHLDA3) has recently been identified as a player in adaptive and maladaptive cellular stress pathways. The outcome of pleckstrin homology-like domain family A—member 3 signalling was shown to vary across different cell types and states. It emerges that its expression and protein level are highly increased in amyotrophic lateral sclerosis (ALS) patient-derived astrocytes. Whether it orchestrates a supportive or detrimental function remains unexplored in the context of neurodegenerative pathologies. To directly address the role of pleckstrin homology-like domain family A—member 3 in healthy and ALS astrocytes, we used overexpression and knockdown strategies. We generated cultures of primary mouse astrocytes and also human astrocytes from control and ALS patient-derived induced pluripotent stem cells harbouring the superoxide dismutase 1 mutation. Then, we assessed astrocyte viability and the impact of their secretome on oxidative stress responses in human stem cell-derived cortical and spinal neuronal cultures. Here, we show that *PHLDA3* overexpression or knockdown in control astrocytes does not significantly affect astrocyte viability or reactive oxygen species production. However, *PHLDA3* knockdown in ALS astrocytes diminishes reactive oxygen species concentrations in their supernatants, indicating that pleckstrin homology-like domain family A—member 3 can facilitate stress responses in cells with altered homeostasis. In support, supernatants of *PHLDA3*-silenced ALS and even control spinal astrocytes with a lower pleckstrin homology-like domain family A—member 3 protein content could prevent sodium arsenite-induced stress granule formation in spinal neurons. Our findings provide evidence that reducing pleckstrin homology-like domain family A—member 3 levels may transform astrocytes into a more neurosupportive state relevant to targeting non-cell autonomous ALS pathology.

## Teaser

Szebényi *et al*. report that the knockdown of pleckstrin homology-like domain family A—member 3 diminishes reactive oxygen species release by human spinal astrocytes harbouring the superoxide dismutase 1 amyotrophic lateral sclerosis mutation and, subsequently, stress response in human spinal neurons. These findings suggest a previously unidentified non-cell autonomous pathogenic process.

## Graphical Abstract

**Graphical Abstract:**
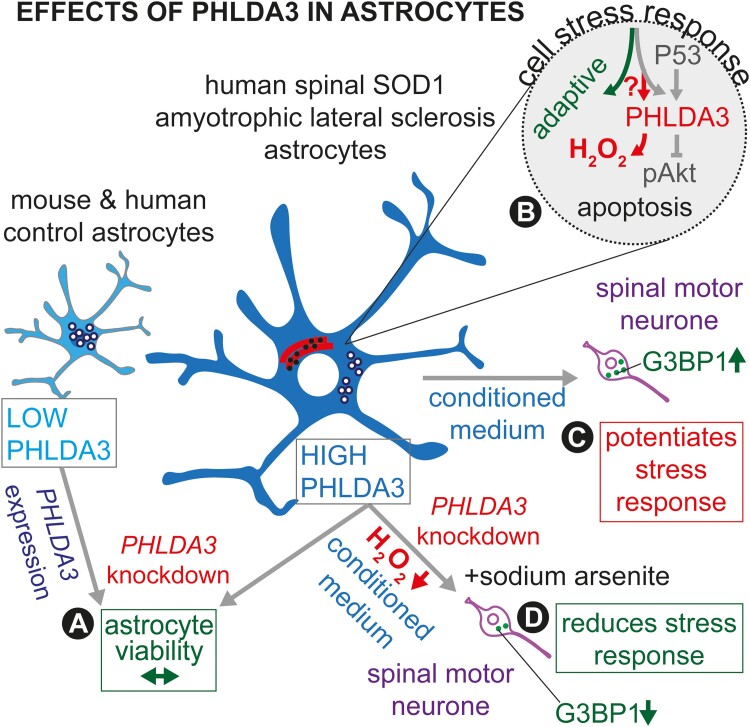

## Introduction

Astrocytes are major contributors to neuronal disturbances in neurodegenerative diseases through exerting both loss of support and gain of toxic functions.^1,2^ These processes attracted therapeutic interest in amyotrophic lateral sclerosis (ALS), an untreatable and fatal disorder with muscle paralysis and variable cognitive impairment in approximately half of the cases. However, the underlying astrocyte-mediated causes are not fully understood.^3^ It emerges that activation of pathways associated with endoplasmic reticulum (ER) stress is an early feature of astrocytes in neurodegeneration and may lead to neuronal vulnerability and synapse loss.^4,5^ The unfolded protein response (UPR) is an important driver in this adaptive mechanism, which can be either supportive or pro-apoptotic, depending on the extent and duration of ER stress.^6^ What guides these responses towards detrimental pathways can vary in different cell types and requires further elucidation.

Our recent study reported a significant increase of a recently identified stress response element, pleckstrin homology-like domain family A—member 3 (PHLDA3), in human superoxide dismutase 1 (SOD1^D90A^)-mutant ALS astrocytes.^7^ It remains unclear whether astrocytic expression of PHLDA3 plays a detrimental or an adaptive protective role, as its function varies in different tissue types. For instance, in ER stress-induced UPR activation in pancreatic cells, PHLDA3 is required for cell survival.^8^ In contrast, the inhibition of PHLDA3 in cardiomyocytes was shown to protect against oxidative stress,^9^ suggesting a harmful role for PHLDA3. In support, the detrimental arm of UPR that activates P53 can lead to PHLDA3-mediated suppression of serine/threonine protein kinase Akt-phosphorylation, affecting cell viability, as seen for hepatocytes.^10,11^

In this work, we addressed two key unresolved issues: how *PHLDA3* expression affects viability in healthy and SOD1-mutant ALS astrocytes and whether it can impact neurons. For this, we employed overexpression and knockdown (KD) strategies, using primary mouse astrocyte cultures, human control and SOD1^D90A^-mutant ALS patient-derived induced pluripotent stem cell (iPSC)-specific spinal astrocytes. In addition, to examine astrocyte-mediated effects, we generated human iPSC (hiPSC)-derived cortical and spinal neurons. Here, we show that raised or low PHLDA3 levels do not affect astrocyte viability, indicated by metabolic assays and analysis of nuclear pyknosis. However, silencing *PHLDA3* expression in human SOD1 ALS spinal astrocytes and even in control astrocytes characterized by lower levels could prevent sodium arsenite (SA)-induced oxidative stress in spinal neurons, measured by the formation of Ras GTPase-activating protein (GAP)-binding protein-1-positive (G3BP1^+^) stress granules (SGs). Our findings indicate that PHLDA3 signalling in astrocytes plays a role in detrimental responses in altered cellular homeostasis, potentially contributing to astrocyte-mediated neuronal injuries in ALS.^12^

## Materials and methods

### Mouse astrocyte cultures

Cell isolation from mouse embryos was performed in accordance with the European Communities Council Directive of 22 September 2010 (2010/63/EU) regarding the use and housing of animals in research and was approved by the Ethics Committee of the Institute of Experimental Medicine CAS and Committee of Czech Academy of Sciences, Prague, Czech Republic, under no. 54/2017. Mouse astrocyte cultures were generated by published protocols resulting in high purity.^13,14^ Briefly, cerebral cortices of newborn pups (P0–P3) of C57BL/6 wild-type mice were isolated and kept on ice in Hibernate E (Thermo Fisher, A1247601). For chemical dissociation, the cortices were first washed with Hank‘s Balanced Salt Solution (HBSS) (Gibco, Thermo Fisher, 14185-045) and treated with 2.5×10^−3^% trypsin (Gibco, Thermo Fisher, 25300-062) in HBSS for 10 min at 37°C. For physical dissociation, the tissue was transferred to a solution containing Hibernate E, 0.4% bovine serum albumin (BSA) (Sigma-Aldrich, A3311) and 100 µg/ml DNase (Sigma-Aldrich, DN25) and triturated with a 1-ml plastic pipette and then with polished Pasteur pipettes (0.3 mm diameter) ten times. The cell suspension was centrifuged for 10 min at 775 RPM before the pellet was resuspended in culture media containing Dulbecco's modified Eagle medium (DMEM) with 1 mM GlutaMAX (Gibco, Thermo Fisher, 10569-010), 10% fetal bovine serum (Gibco, Thermo Fisher, A5256801) and 1% penicillin/streptomycin (Gibco, Thermo Fisher, 15140-122). The cells from two cortices were then plated in a T25 culture flask (Thermo Fisher) and cultured at 37°C and 5% CO_2_ until confluency with media changes at every 2–3 days for 7–9 days. Subsequently, the flasks were shaken at 260 RPM at 37°C overnight, followed by washes with PBS to remove the detached cells (microglia and neural progenitors), and the cells were replated and cultured for another 7–9 days until near confluency, at which point this cycle was repeated once again. Astrocytes were plated onto poly-D-lysine (PDL)-coated coverslips (5000 cells per well) in 24-well plates for biological assays.

### Human astrocyte cultures

The generation of hiPSC-derived glial progenitors and astrocytes was based on published protocols^7,15^ and commercially available cell lines (control: EBiSC WTSIi097-A, MTA Ref_RG92224 for A.L.; SOD1^D90A^ ALS: NINDS ND35664, MTA Ref_G107192 for A.L.). hiPSCs were differentiated towards neural fate by applying a chemically defined medium containing 1 μM dorsomorphin (Tocris Bioscience, 3093/10), 2 μM SB431542 (Tocris Bioscience, 1614) and 3 μM CHIR99021 (Tocris Bioscience, 4423) for 7 days. Then, neural precursors were patterned for an additional 7 days with 0.5 μM retinoic acid (Abcam, ab120728) and 1 μM purmorphamine (Tocris Bioscience, 4551), followed by a 4-day treatment with 0.1 μM purmorphamine which was followed by a propagation phase (>60 days) with 10 ng ml^−1^ fibroblast growth factor (FGF)-2 (Peprotech, 100-18B-50). Next, glial progenitor cells were differentiated in N2B27 medium supplemented with bone morphogenetic protein (BMP)-4 (10 ng ml^−1^, Peprotech, 120-05-5) and leukaemia inhibitory factor (LIF, 10 ng ml^−1^, Peprotech, 300-05-5) for 30 days, followed by an additional maturation phase of 30 days when cells were only maintained in N2B27.

### Human i^3^N cortical neuronal differentiation

i^3^N neurons (gift from Prof. Evan Reid) were differentiated via induced expression of the neurogenin-2 transcription factor as previously described.^16^ In brief, hiPSCs were grown in Stemflex media (Thermo Fisher Scientific, A3349401) on six-well plates coated with Geltrex (Thermo Fisher Scientific, A1413302). Once the cells were 80% confluent, they were washed with PBS -/- and treated with accutase (Sigma-Aldrich, A6964-100 ML) for 5 min at 37°C to create a single cell suspension. Next, the cells were transferred to a 10-cm Geltrex-coated dish and differentiation was started by the addition of doxycycline in culture media containing DMEM/F12 (Thermo Fisher Scientific, 31331028), N2 (Thermo Fisher Scientific, 17502048), non-essential amino acid (NEAA) solution (Thermo Fisher Scientific, 11140035) and Glutamax for 4 days. The cells were then replated on coverslips coated with polyethyleneimine (PEI) and Geltrex (80k/coverslip) and grown in neuronal media [Brainphys (Stem Cell Technologies, 05790), B27 (Thermo Fisher Scientific, 17504044), neurotrophin-3 (NT3, Peprotech, 450-03), brain-derived neurotrophic factor (BDNF) (Peprotech, 450-02-10ug) and Laminin (Gibco, 23017015)] for 14 days with half media changes every 3 days.

### Human spinal motor neuron differentiation

Human spinal motor neurons were differentiated from hiPSCs, based on a previously published protocol.^17^ Briefly, 80% confluent monolayers of WTSli097-A hiPSCs were switched to N2B27 medium supplemented with 1 μM dorsomorphin (Millipore), 2 µM SB-431542 and 3 µM CHIR99021. On Day 8, the cells were dissociated with accutase and plated onto Geltrex-coated culture dishes in N2B27 medium supplemented with 500 nM retinoic acid (Abcam, ab120728) and 1 μM purmorphamine (Tocris Biosciences, 4551/10). On Day 14, the cells were detached with 0.5 mM EDTA and transferred to an uncoated flask in fresh N2B27 medium supplemented with retinoic acid, purmorphamine and 10 µM Y-27632 (Tocris Biosciences, 1254/10). From Day 18, N2B27 medium was supplemented with 100 nM Compound-E (Abcam, ab142164). After 72 h, the neurospheres were dissociated with papain, and the cells were plated on 96-well plates in N2B27 medium supplemented with 10 µM Y-27632 and Compound-E (Abcam, ab142164). After 7 days, the cells were switched into N2B27 with glial cell line-derived neurotrophic factor (GDNF) (Peprotech, 450-10-10) and BDNF (Peprotech, 450-02-10) and cultured until 50 days *in vitro* (DIV) before the experiments.

### PHLDA3-green fluorescent protein and green fluorescent protein plasmid transfections

Mouse or hiPSC-derived astrocytes seeded onto poly-L-ornithine (Sigma-Aldrich)-coated glass coverslips in a 24-well plate were transfected with either a cytomegalovirus promoter (pCMV) containing ‘pCMV-green fluorescent protein (GFP)’ or ‘pCMV-empty’ control adeno-associated virus (AAV) vector-expressing plasmids (AAV-CMV-eGFP; Addgene 193022 and backbone, provided by Bart Nieuwenhuis/James Fawcett) or a pCMV-PHLDA3-GFP plasmid (pCMV6-AC-*PHLDA3-GFP*; Origene, RG206751), using Lipofectamine 2000 (Thermo Fisher) according to manufacturer's instructions. Transfection optimization was carried out, using plasmids at 0.5 and 1 µg doses and lipofectamine at 0.5, 1, 2 and 3 µl volumes per culture (0.5 ml media per well). Astrocytes were transfected for 5 h and used at 48 h posttransfection for experimental assays.

### PHLDA3 siRNA-based KD strategy

For *PHLDA3*-KD experiments, the SMARTpool of ON-TARGETplus PHLDA3 siRNA (80 nM final concentration, Dharmacon, L-020169-00-0005) and for the scrambled (scr) siRNA controls, the ON-TARGETplus Non-targeting Control Pool (80 nM final concentration, Dharmacon, d-001810-10-05) were used. Human astrocyte cultures were incubated with siRNAs in combination with the Lipofectamine™ RNAiMAX Transfection Reagent (Thermo Fisher Scientific, 13778100), according to the manufacturer's protocols for 72 h, and the cells were used for assays within 72 h posttransfection.

### Cell viability and reactive oxygen species assays

The alamarBlue™ (Thermo Fisher Scientific, DAL1025) or PrestoBlue™ (Thermo Fisher Scientific, A13262) assays were used for astrocyte viability assessments. In addition, cell death was quantified manually by counting DAPI-stained pyknotic nuclei in GFP^+^ transfected astrocytes and NeuN^+^ neurons. The reactive oxygen species (ROS)-Glo H_2_O_2_ assay (Promega, G8820) was used to detect ROS levels in astrocyte-conditioned media (ACM), indicated by the H_2_O_2_ luminescence signal which was measured by the Promega GloMAX Plate reader.

### Astrocyte-conditioned media and SA treatment

ACM were collected overnight from control or SOD1 ALS astrocytes 48 h after transfection with overexpression plasmids, siRNA or vehicle or without transfection. For the treatment of neurons with ACM, half of the neuronal culture media (250 μl) was substituted with warm ACM derived from control or SOD1 ALS astrocytes (previously treated with scr siRNA or PHLDA3 siRNA) for 3 h prior to the treatment of neurons with SA in the same media. Neurons were treated by SA at the final concentration of 0.5 mM for 1 h at 37°C before they were used in experimental assays.

### Western blots

Mouse and human astrocyte protein samples were prepared using radio-immunoprecipiation assay (RIPA) lysis buffer (Sigma-Aldrich, R0278) and supplemented with protease inhibitors (Pierce Protease Inhibitor Mini Tablets, Thermo Fisher Scientific, 31462 or Roche PhosphoSTOP™ EASYpack, Sigma-Aldrich, 4906845001, respectively) and phosphatase inhibitors (Pierce Phosphatase Inhibitor Mini Tablets, Thermo Fisher Scientific, A32957). Protein concentrations in the supernatants were measured using the Pierce bicinchoninic acid (BCA) assay (Thermo Fisher Scientific, 23227). 30 μg of protein was resolved by SDS–PAGE (4–15% Mini-PROTEAN® TGX™ Precast Protein Gels, Bio-Rad or NuPAGE™ 12%, Bis–Tris, 1.0 mm, Mini Protein Gels, Thermo Fisher Scientific, NP0341BOX) and then transferred onto polyvinylidene difluoride (PVDF) membranes (0.45 μm pore size; Life Technologies). Membranes were blocked using either 5% BSA (Sigma-Aldrich) or 5% dried milk (Cell Signaling) in Tris-buffered saline/Tween-20 (TBST), depending on antibody requirements. Membranes were incubated overnight with the primary antibodies in five times-diluted blocking solution at 4°C with gentle agitation on a shaker, except for the directly labelled β-actin antibody, which was applied only for 1 h. Then, horseradish peroxidase (HRP)-conjugated secondary antibodies were applied in TBST for 2 h at room temperature (RT) (see Supplementary Table 1 for antibody details). HRP was visualized using the Clarity™ Western ECL Substrate (Bio-Rad) or the enhanced chemiluminescence system (GE Healthcare, RPN2232). All uncropped scans of western blots (WBs) are included in Supplementary Figs. 1–6.

### Immunocytochemistry

Cultured cells were fixed using 4% PFA in PBS and immunostained by standard protocols.^7^ In brief, mouse and human cells were blocked and permeabilized in PBS containing 10% goat serum and 0.4% Triton-X for 60 min or 0.3% Triton-X for 45 min, respectively, at RT. Mouse cells were incubated overnight with primary antibodies at 4°C, followed by secondary antibodies for 60 min in 2% goat serum/0.1% Triton-X in PBS at RT. For human cells, primary and secondary antibodies were applied for 90 min in 3% goat serum/0.1% Triton-X in PBS and 60 min in PBS, respectively, at RT (see Supplementary Table 1 for antibody details). For immunolabelling with the goat anti-choline acetyltransferase (ChAT) antibody, goat serum was replaced by normal donkey serum (Abcam, ab7475) in each step. Nuclei were stained using DAPI diluted 1:3000 in PBS. The cells were then washed three times with PBS, and the coverslips were mounted with the FluorSave (Merck Millipore, 345789) mounting reagent.

### Image processing and cell counts

For mouse cells, confocal microscopy images were captured by a Zeiss LSM 880 inverted microscope (20× and 40× magnification, 2048×2048 pixel resolution). For human cells, confocal microscopy imaging was performed using the Leica DMI 4000 B microscope (63× magnification, 1.5 zoom, bidirectional scanning, 512×512 and 2048×2048 pixel resolution) and the LAS-AF software (Leica, version 2.7.3.9723). Z-stack images were obtained by capturing confocal planes at 0.5-μm intervals. Single-plane and maximum-intensity z-projection images were created in Fiji (ImageJ version 2.3.0) software and used for illustrations.^18^ Counts of pyknotic nuclei were performed manually. For quantifying the nucleus-to-cytoplasm shift of PHLDA3 immunoreactivity (IR), area- and shape-independent mean fractional intensity (MeanFrac) measurements were taken, and for SG quantification, G3BP1^+^ granule and NeuN^+^ cell counts were performed using CellProfiler.^19^ Chemiluminescence in WB membranes was imaged using the Alliance 4.7 CCD Image System (UVITEC). Quantification of band densities was performed in Fiji by standard methods.^5^ For data comparison, band density values were normalized to corresponding β-actin^+^ bands and a control sample within the same blot. Camera exposure and gain were kept the same while collecting images, and analyses were performed using the non-modified original images. The recommended guidelines were followed to illustrate immunofluorescence and microscopic images. Pseudo-colours in images were rendered in Fiji for multicolour visualization in images. Images were minimally processed in Fiji or Adobe Photoshop (v21.0.3), which included uniform changes in gain parameters when clear views were obscured in merged panels and applied equally to images that were directly compared. For WBs, blot images were cropped for focused views, leaving a six-band width. All original uncropped WB images are presented in the Supplementary material. Figure assembly and drawings were performed in Adobe Illustrator (v24.0.3). The use of schematic images complies with the content licenses of pixabay and Motifolio (2011).

### Statistical analysis

Statistical test details are included in Supplementary Table 2. Briefly, sample identifiers were blinded for the observers. Statistical tests were only run using data from independent biological repeats (at least three independent cultures/transfections). For illustration, data points from each technical repeat were also plotted from repeated experiments where applicable. The GraphPad Prism v.8.0 software was used for normality tests, statistical analyses and illustrations. For the comparison of two groups, unpaired two-tailed *t*-test was used, and for multiple comparisons, two-tailed one-way ANOVA was used with Dunnett's or Tukey's *post hoc* test in the case of overall statistical significance. Statistical significance was accepted at *P* < 0.05. The exact sample sizes, *P*-values and type of tests were indicated in the figures and legends.

## Results

### *PHLDA3* overexpression or KD does not influence viability in control astrocytes

**Figure 1:**
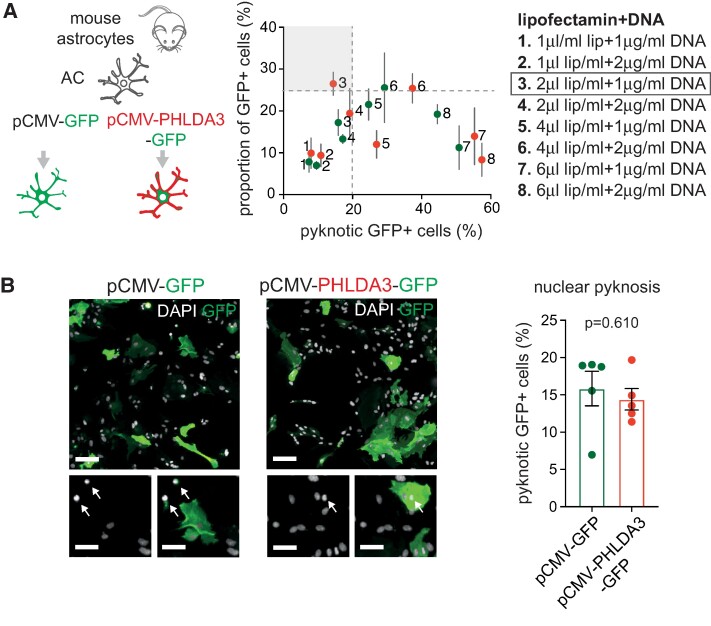
***PHLDA3* overexpression does not induce mouse astrocyte nuclear pyknosis.** (**A**) Schematic of astrocyte transfection strategy (left), using the pCMV-GFP or pCMV-PHLDA3-GFP expression plasmid. Assays were evaluated 48 h posttransfection. Graph (right) shows the percentages of GFP^+^-transfected cells against the percentage of pyknotic nuclei out of total nuclei counts, using various concentration ratios of Lipofectamine 2000 and plasmid DNA. Data expressed as mean ± SD. (**B**) Representative immunofluorescence images of transfected astrocytes stained for GFP (arrows indicate pyknotic cells). Graph demonstrates the percentage of pyknotic GFP^+^ cells determined at the optimal lipofectamine (1 μl/ml)/DNA (2 μg/ml) concentration. Scale bars: 60 µm (30 µm for insets). *n* = 5 independently transfected cultures; unpaired two-tailed *t*-test; data are expressed as mean ± SEM. For further statistical details, see Supplementary Table 2.

To investigate the role of increased *PHLDA3* expression in astrocytes, we initially used readily available purified C57Bl/6 wild-type mouse astrocyte cultures transfected with either a pCMV-PHLDA3-GFP or a pCMV-GFP-expressing control plasmid (Fig. 1A). Astrocyte cultures were subjected to assays at 48 h posttransfection. First, we optimized transfection efficiencies while examining the effect of *PHLDA3* expression on astrocyte death by analysing nuclear pyknosis. The highest transfection efficiency was achieved at 26.49%, associated with limited pyknosis at 2 μl/ml lipofectamine and 1 μg/ml DNA final concentrations for the *PHLDA3* overexpression vector (Fig. 1A, B). The proportion of GFP^+^ pyknotic cells between the PHLDA3-GFP and GFP-transfected cell groups were comparable (*P* = 0.61), suggesting that the increase of PHLDA3 in astrocyte cultures does not induce cell death at this titre (Fig. 1B).

**Figure 2:**
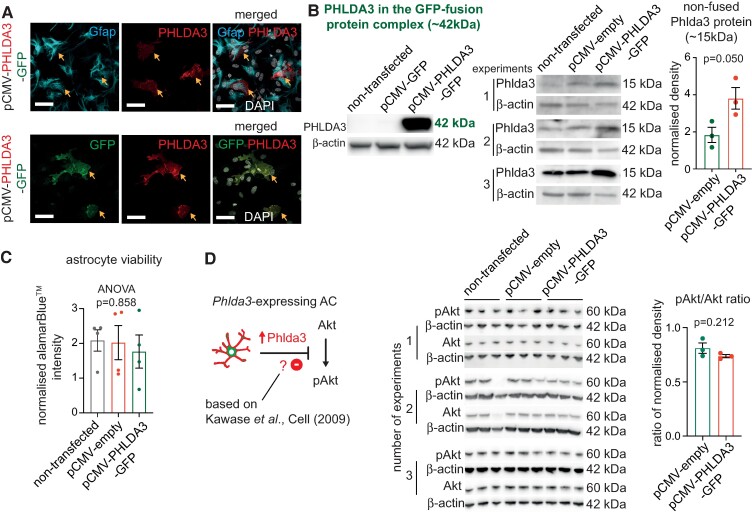
***PHLDA3* overexpression does not alter mouse astrocyte (AC) viability and pAkt/Akt protein-level ratios**. (**A**) Representative immunofluorescence images showing PHLDA3^+^ and GFAP^+^ (upper panel) or GFP^+^ double-labelled cells (lower panel) in purified AC cultures transfected with pCMV-PHLDA3-GFP. Scale bars: 40 µm. (**B**) WBs displaying PHLDA3 and β-actin^+^ bands for lysates of non-transfected or transfected ACs with the pCMV-GFP or pCMV-empty control or the pCMV-PHLDA3-GFP vector. PHLDA3 labelling corresponds with the GFP-fusion protein (∼42 kDa, left-sided panel) and with the non-fused or endogenous protein (15 kDa, right-sided panel). Graph represents the Phlda3^+^ band densities normalized to the non-transfected group and to β-actin in each blot. *n* = 3 independent cultures; unpaired two-tailed *t*-test; data are expressed as mean ± SEM. (**C**) Graph represents normalized signal intensities in the alamarBlue™ viability assay. *n* = 4 independent cultures; unpaired two-tailed *t*-test; data are expressed as mean ± SEM. (**D**) Schematic of a previously demonstrated signalling route for Phlda3. WBs demonstrating immunoreactive bands for pAkt, Akt and β-actin for lysates of non-transfected or transfected ACs with the pCMV-empty or the pCMV-PHLDA3-GFP vector. Graph represents the ratios of pAkt/Akt band densities normalized to the non-transfected group and to β-actin in each blot. *n* = 3 independent cultures; unpaired two-tailed *t*-test; data are expressed as mean ± SEM. For further statistical details, see Supplementary Table 2. For full WB scans, see Supplementary Figs. 1 and 2.

Next, we verified *PHLDA3* expression using immunocytochemistry and immunoblots of astrocyte cultures transfected with either the pCMV-GFP empty or the pCMV-PHLDA3-GFP vector. PHLDA3 IR was abundant in transfected astrocytes, which also displayed glial fibrillary acidic protein (GFAP) and GFP IR (Fig. 2A). Notably, GFP-negative cells that were not transfected by the overexpression plasmid did not show strong Phlda3 IR (Fig. 2A). WBs demonstrated strong immunoreactive bands for PHLDA3 at ∼42 kDa, corresponding with the PHLDA3-GFP-fusion protein in astrocytes transfected with the pCMV-PHLDA3-GFP vector. In comparison, no visible bands were detected in the non-transfected or pCMV-GFP-transfected control samples that lacked the fusion protein (Fig. 2B). *PHLDA3*-*GFP*-overexpressing astrocyte cultures also showed a 1.76-fold increase in band densities for Phlda3 at 15 kDa compared to the empty vector-transfected group, indicating fusion protein dissociation or an increase in endogenous protein levels (Fig. 2B).

The confirmation of raised PHLDA3 protein levels in astrocytes allowed us to examine its broader impact on cell viability via a metabolic assay and downstream survival signalling. The alamarBlue™ viability test revealed no differences between non-transfected, pCMV-empty and pCMV-PHLDA3-GFP-transfected astrocytes (ANOVA *P* = 0.858; Fig. 2C). Since Phlda3 is known to repress the phosphorylation of Akt,^11^ a survival signal, we tested whether it fails in healthy astrocytes, given that their viability was unaffected. WBs revealed comparable ratios of normalized phosphorylated Akt (pAkt)/Akt band densities between pCMV-PHLDA3-GFP and pCMV-empty plasmid-transfected astrocyte cultures (*P* = 0.212), indicating no differences in their viability (Fig. 2D).

**Figure 3:**
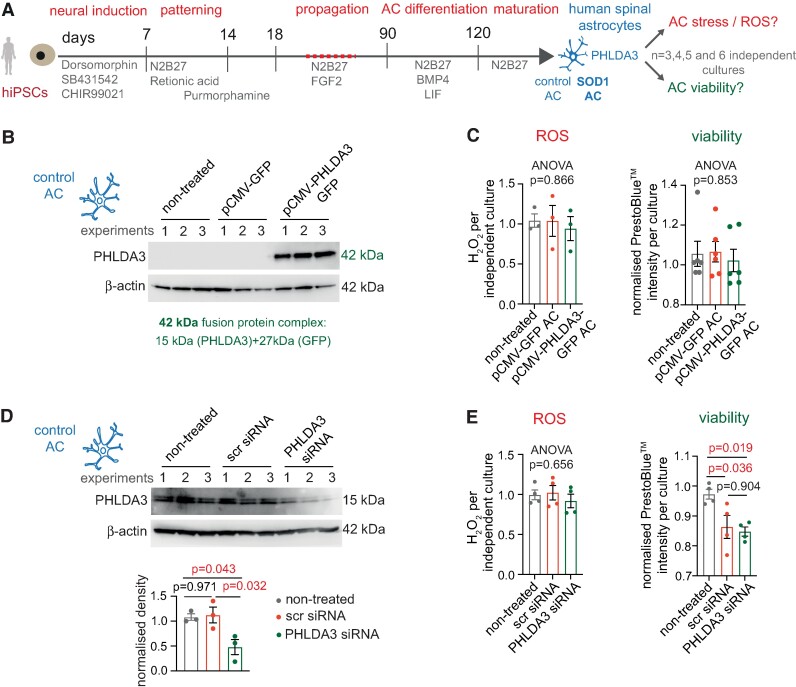
**PHLDA3 protein-level changes do not influence human control astrocyte (AC) ROS production or viability**. (**A**) Schematic illustration of iPSC-derived spinal AC generation. (**B**) WBs displaying PHLDA3 and β-actin-positive bands for lysates of non-transfected or transfected control ACs with the pCMV-GFP control or the pCMV-PHLDA3-GFP vector. PHLDA3 labelling corresponds with the GFP-fusion protein (∼42 kDa). *n* = 3 independent cultures. Assays were performed 48 posttransfection. (**C**) Graphs represent normalized ROS levels measured by the ROS-Glo assay (left) and PrestoBlue™ intensity values (right) per culture. *n* = 3 and 6 independent cultures, respectively; one-way ANOVA. (**D**) WBs displaying bands for PHLDA3 and β-actin for protein lysates of non-treated, scrambled (scr) siRNA- and PHLDA3 siRNA-treated control ACs. Graph illustrates normalized band densities for each group. *n* = 3 independent cultures; one-way ANOVA with Dunnet's *post hoc* test. Assays were performed 72 h posttransfection. (**E**) Graphs represent normalized ROS levels (left) measured by the ROS-Glo assay and PrestoBlue™ intensity values (right) per culture of non-treated, scr siRNA- and PHLDA3 siRNA-treated control ACs. *n* = 4 independent cultures; one-way ANOVA with overall *P*-value (left) and Tukey's *post hoc* test (right). Data are expressed as mean ± SEM for all graphs. For further statistical details, see Supplementary Table 2. For full WB scans, see Supplementary Figs. 3 and 4.

We then perturbed PHLDA3 levels in hiPSC-derived spinal astrocytes to examine how this affects their survival^15^ (Fig. 3A–E). Since PHLDA3 inhibition was shown to reduce oxidative stress in cardiomyocytes,^9^ we tested whether PHLDA3 level changes impact the release of ROS and cell viability, using ROS and PrestoBlue™ assays, respectively. The efficient plasmid-based overexpression or siRNA-based KD in control astrocytes was confirmed by quantified immunoblots at 48 or 72 h posttransfection for the cell culture assays, respectively (Fig. 3B, D). The latter also demonstrated double bands at ∼15 kDa, indicating the functional protein and a possible isoform often seen in both mouse and human samples.^20,21^ Neither overexpression nor KD altered ROS levels or viability (Fig. 3B–E). Altogether, our results suggest that PHLDA3 alone does not significantly impede the survival of human spinal astrocytes.

### *PHLDA3* KD leads to reduced ROS levels in human SOD1 ALS astrocytes

**Figure 4:**
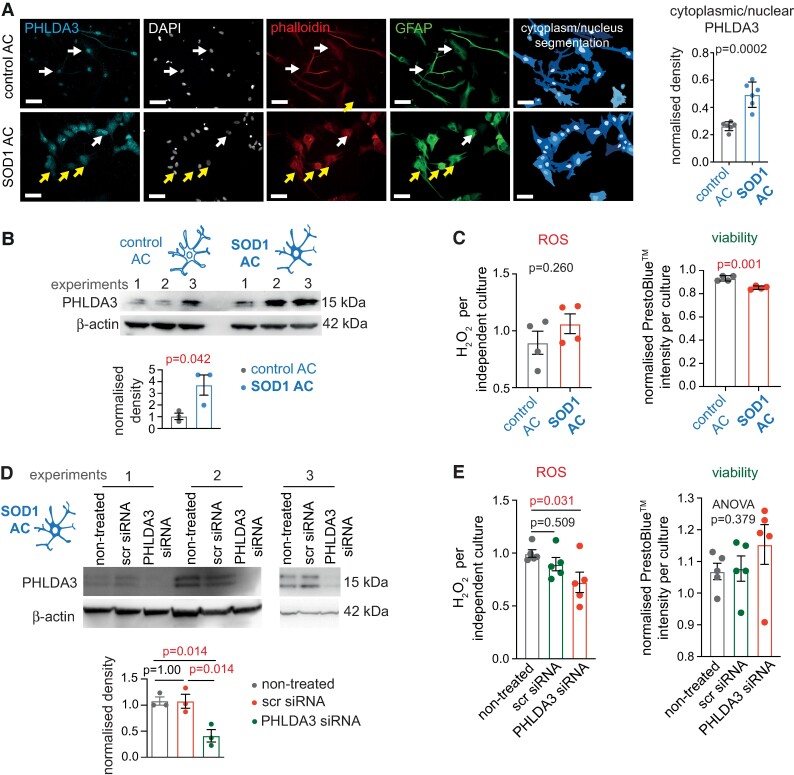
**PHLDA3-KD diminishes ROS production in human SOD1 ALS astrocytes (ACs)**. (**A**) Representative immunofluorescence images of control and SOD1 ALS ACs, demonstrating nuclear (arrows in upper panel) and cytoplasmic (arrows in lower panel) distribution of PHLDA3 IR in GFAP^+^ ACs. Digital images (right) represent area segmentations for cytoplasmic and nuclear PHLDA3 IR measurements based on DAPI and phalloidin staining. Scale bars: 40 µm. Graphs show the ratio of cytoplasmic/nuclear density of PHLDA3 IR in control and SOD1 ALS AC. *n* = 6 cultures; unpaired two-tailed *t*-test. (**B**) WBs displaying bands for PHLDA3 and β-actin for protein lysates of control and SOD1 ALS ACs. Graph illustrates normalized band densities for each group. *n* = 3 independent cultures; unpaired two-tailed *t*-test. (**C**) Graphs represent normalized ROS levels (left) measured by the ROS-Glo assay and PrestoBlue™ intensity values (right) per culture of control and SOD1 ALS ACs. *n* = 4 independent cultures; unpaired two-tailed *t*-test. (**D**) WBs displaying bands for PHLDA3 and β-actin for protein lysates of non-treated, scr siRNA- and PHLDA3 siRNA-treated SOD1 ALS ACs. Graph illustrates normalized band densities for each group. *n* = 3 independent cultures; one-way ANOVA with Dunnet's *post hoc* test. (**E**) Graphs represent normalized ROS levels (left) measured by the ROS-Glo assay and PrestoBlue™ intensity values (right) per culture of non-treated, scr siRNA- and PHLDA3 siRNA-treated SOD1 ALS ACs. *n* = 5 independent cultures; one-way ANOVA with Dunnett's *post hoc* test (left) and overall ANOVA *P*-value (right). Data are expressed as mean ± SEM for all graphs. For further statistical details, see Supplementary Table 2. For full WB scans, see Supplementary Figs. 5 and 6.

Next, we addressed whether PHLDA3 could affect human astrocyte ROS production and viability in pathological conditions. For this, we used the same human SOD1^D90A^-mutant ALS patient-specific iPSC line to derive spinal astrocytes^15^ (onwards ALS astrocytes), in which the increased PHLDA3 protein levels and predominant cytoplasmic protein distribution were originally observed by mass spectrometry and immunolabelling, respectively.^7^ First, we confirmed the PHLDA3 distribution pattern required for cytoplasmic signalling and the cellular protein abundance. We performed PHLDA3/GFAP co-immunolabelling in control and ALS astrocyte cultures and assessed the intracellular localization of PHLDA3, using CellProfiler. The results showed a significant (*P* = 0.0002) 1.88-fold increase in the nucleus-to-cytoplasmic shift of PHLDA3 IR based on mean fractional intensity measurements which are independent of the cell shape and area of astrocytes (Fig. 4A). WBs also confirmed the increased overall PHLDA3 levels in ALS versus control astrocytes (Fig. 4B). These observations were associated with a statistically comparable ACM ROS concentration between control and ALS astrocytes (*P* = 0.258), but the latter displayed reduced viability by 9.49% (Fig. 4C), which could have masked the observations on ALS astrocyte-produced ROS. Therefore, we knocked down *PHLDA3* expression to examine its direct impact on ROS production by ALS astrocytes, which was confirmed by quantified immunoblots (Fig. 4D). The reduction of PHLDA3 protein levels in ALS ACM led to a 1.24-fold (*P* = 0.031) decrease in ROS levels when compared with ACM of non-treated ALS astrocytes that were comparable to scr siRNA-treated ALS astrocytes (*P* = 0.51), while their viability was unaffected (ANOVA *P* = 0.379; Fig. 4E). Our findings indicate that PHLDA3 can potentiate homeostatic disturbances in ALS astrocytes, which may influence their supportive state towards neurons.

### PHLDA3 inhibition in human astrocytes prevents SA-induced spinal neuronal stress

**Figure 5:**
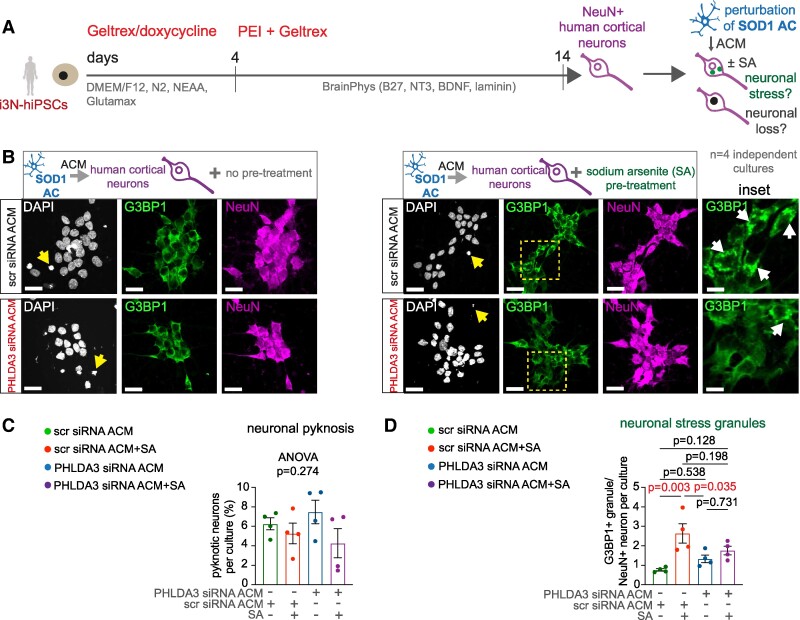
**SA-induced human cortical neuronal SG formation is diminished by ACM produced by PHLDA3-KD ALS astrocytes (ACs)**. (**A**) Schematic illustration of hiPSC-derived cortical i3N neuron culture generation and experimental strategy. (**B**) Representative immunofluorescence images of human i3N neurons with or without SA treatment for 1 h following incubation for 3 h in ACM of scrambled (scr) siRNA- or PHLDA3 siRNA-transfected human SOD1 ALS ACs, displaying stress granule marker (G3BP1) and NeuN IR and DAPI staining (arrows indicate pyknotic nuclei). Scale bars: 20 μm. Insets represent magnified G3BP1 immunoreactive areas (dashed yellow squares) and SG deposits (arrows). Scale bars: 50 μm. (**C**) Graphs represent the percentage of pyknosis in scr siRNA-ACM- and PHLDA3 siRNA-ACM-treated NeuN^+^ neurons per culture. *n* = 4 independent cultures; one-way ANOVA. (**D**) Graphs represent the number of G3BP1^+^ SGs in cultures of scr siRNA-ACM- and PHLDA3 siRNA-ACM-treated neurons in NeuN^+^ neurons per culture. *n* = 4 independent cultures; one-way ANOVA with Tukey's *post hoc* test. Data are expressed as mean ± SEM for all graphs. For further statistical details, see Supplementary Table 2.

We assessed whether reducing PHLDA3 levels in human spinal ALS astrocytes could reduce neuronal stress responses. In an initial experiment, we generated cultures of human cortical neurons (i^3^N), which can be rapidly established using previously published protocols^16^ and treated the cultures with ACM samples at 14 DIV for 3 h before neuronal oxidative stress was induced by SA (Fig. 5A). For this, ACM was collected from human SOD1-mutant astrocytes treated with scrambled siRNA (scr siRNA ACM) or siRNA to knockdown *PHLDA3* (siRNA PHLDA3-KD ACM). Then, we evaluated the neuronal oxidative stress by assessing adaptive SG accumulation and also cell death by quantifying pyknosis in DAPI-stained nuclei (Fig. 5B–D). SGs in neurons were visualized by double immunolabelling for G3BP1, a reliable SG marker^22^ and for NeuN, a mature neuronal marker. Although neuronal pyknosis was not affected by SA (Fig. 5B, C), cortical neurons showed a significant 3.43-fold increase in G3BP1^+^ SG density when exposed to SA in the presence of scr siRNA ACM (Fig. 5B, D). PHLDA3 siRNA ACM diminished SA-induced increase in SG formation (1.76 ± 0.22/cell) in cortical neurons; however, the extent of this decrease was not significant when compared with the scr siRNA ACM-treated group (2.64 ± 0.5/cell, *P* = 0.198, Fig. 5D), indicating only a partial response.

**Figure 6:**
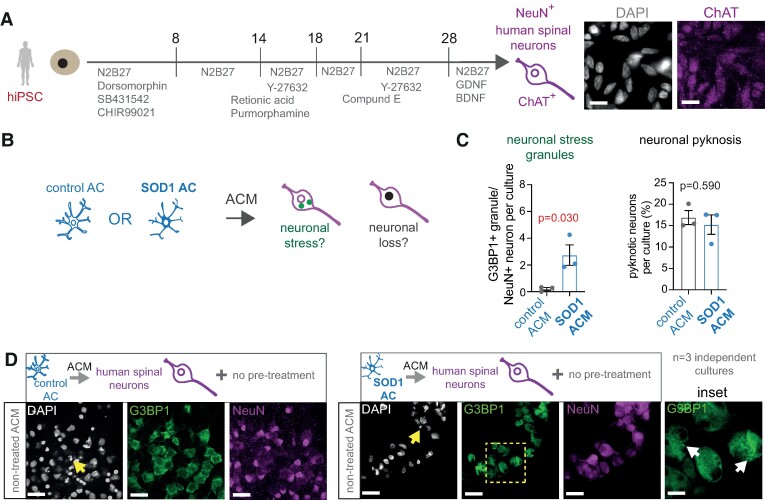
**Human spinal neuronal SG formation is increased by ACM produced by human spinal SOD1 ALS astrocytes (ACs)**. (**A**) Schematic illustration of hiPSC-derived spinal neuron culture generation. Representative immunofluorescence images show ChAT^+^ positive spinal neurons with corresponding DAPI staining. Scale bars: 10 μm. (**B**) Experimental strategy. (**C**) Graphs represent the percentage of G3BP1^+^ SGs (left) and nuclear pyknosis (right) in NeuN^+^ neurons per culture treated with either control or SOD1 ALS ACM. *n* = 3 independent cultures; unpaired two-tailed *t*-test. (**D**) Representative immunofluorescence images of hiPSC-derived spinal neurons with incubation in either control or SOD1 ALS ACM, displaying SG marker (G3BP1) and NeuN IR and DAPI staining (arrows represent pyknotic nuclei). Scale bars: 40 μm. Inset represents magnified G3BP1 IR areas (dashed square) and SG deposits (arrows). Scale bar: 100 μm. Data are expressed as mean ± SEM for all graphs. For further statistical details, see Supplementary Table 2.

**Figure 7:**
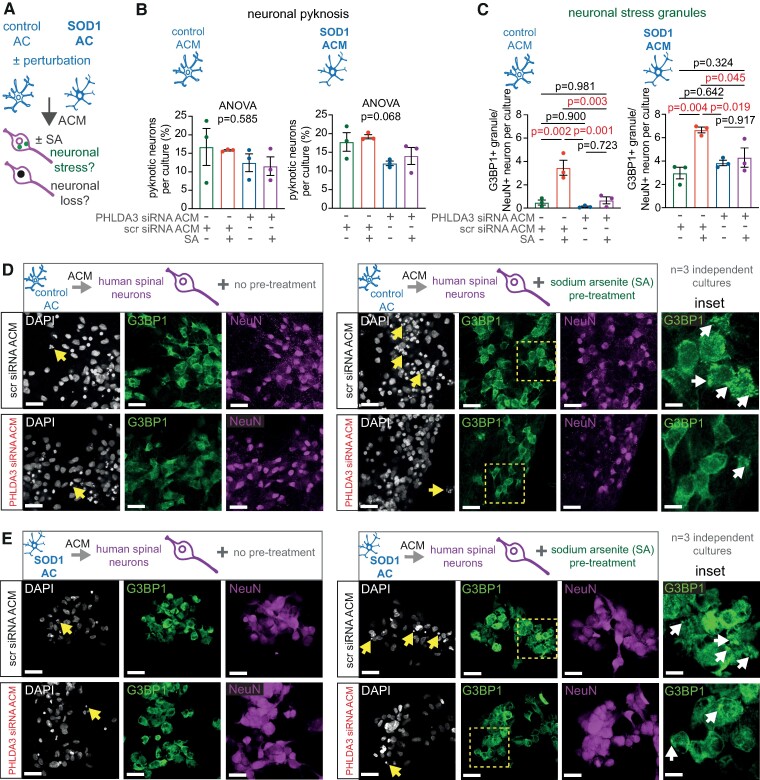
**SA-induced human spinal neuronal SG formation is reduced by ACM produced by human spinal PHLDA3-KD astrocytes (ACs)**. (**A**) Schematic illustration of experimental strategy. Spinal neurons were exposed to SA treatment or vehicle for 1 h following incubation for 3 h in ACM. Graphs represent the percentage of neuronal pyknosis (**B**) and G3BP1^+^ SGs (**C**) in scrambled (scr) siRNA-ACM- and PHLDA3 siRNA-ACM-treated NeuN^+^ neurons per culture. *n* = 3 independent cultures; one-way ANOVA with Tukey's *post hoc* test (for C). Representative immunofluorescence images of hiPSC-derived spinal neurons with or without SA treatment for 1 h following incubation for 3 h in ACM of scr siRNA- or PHLDA3 siRNA-transfected control (**D**) and SOD1 ALS ACs (**E**), displaying SG marker (G3BP1) and NeuN IR and DAPI staining (arrows represent pyknotic nuclei). Scale bars: 40 μm. Insets represent magnified G3BP1 IR areas (dashed squares) and SG deposits (arrows). Scale bars: 100 μm. Data are expressed as mean ± SEM for all graphs. For further statistical details, see Supplementary Table 2.

We then tested whether lower PHLDA3 levels in human spinal astrocytes can prevent stress responses in neurons with the same regional identity. For this, we generated spinal NeuN/ChAT + neurons from hiPSCs using standard protocols^16^ (Fig. 6A). Initially, we examined whether the secretome of control astrocytes displaying lower PHLDA3 levels induced less neuronal SG accumulation than that seen for ALS astrocytes, while no differences were found in cell death, indicated by the comparable nuclear pyknosis (Fig. 6B–D). ACM from non-treated control astrocytes caused significantly lower SG formation (0.21 ± 0.11/cell, *P* = 0.030) when compared with ALS astrocytes (2.74 ± 0.76/cell), and it remained negligible regardless of pretreatment with scr siRNA or PHLDA3 siRNA (Figs. 6C, D and 7A–E). Again, nuclear pyknosis remained comparable within the groups (Figs. 6C and 7B, D, E). Treatment with control or ALS astrocyte ACM allowed SA-induced increases in SG density, albeit it reached greater values in the presence of SOD1 (6.66 ± 0.25/cell) than control scr siRNA ACM (3.46 ± 0.64/cell). However, the siRNA-based reduction of PHLDA3 levels in control and ALS astrocytes both lowered the SA-induced neuronal SG accumulation to the non-SA-induced baseline values (Fig. 7C–E). Our results indicate that although the higher PHLDA3 abundance in ALS versus control astrocytes allows a greater ACM-induced neuronal stress response, the reduction of PHLDA3 protein levels in both control and spinal ALS astrocytes can lead to a beneficial effect in spinal neuronal stress.

## Discussion

Our work provides evidence that increased *PHLDA3* expression alone does not influence astrocyte viability. However, the reduction of raised PHLDA3 levels associated with neurodegenerative cues could improve astrocytic homeostasis disturbances, such as ROS production. Lowering PHLDA3 levels in human SOD1 ALS spinal astrocytes and even in control astrocytes characterized by lower PHLDA3 content in culture had a protective effect on spinal neuronal stress. This suggests a contributory role of PHLDA3 in astrocyte-mediated cellular disturbances, which may be enhanced in the context of neurodegenerative conditions such as ALS.

The GFP-tagged plasmid-based transfection strategy combined with nuclear labelling and immunostaining allowed us to examine the viability directly in those astrocytes in which PHLDA3 levels were raised. Our findings suggest that the impact of PHLDA3 on cell survival is either redundant in astrocytes or depends on the cell state. In support, PHLDA3-mediated cellular changes were mainly observed in the context of ER or oxidative stress in previous studies.^8-10^ Altogether, these results suggest that detrimental PHLDA3 signalling becomes relevant when cell homeostasis is already altered.

To define the role of PHLDA3 in neurodegenerative pathology, we used human spinal SOD1^D90A^-mutant ALS patient-specific iPSC-derived astrocytes that were previously shown to display signs for ER stress, associated with increased *PHLDA3* expression and protein levels.^7^ The observed cytoplasmic versus nuclear abundance of PHLDA3 IR in ALS astrocytes is aligned with previous findings^7^ and suggests downstream cytoplasmic signalling activation.^23^ ER-stress-induced UPR, a previously described trigger for PHLDA3-mediated pathways,^10^ may result in different outcomes depending on which signalling arm dominates the response.^24^ For instance, phosphorylation of eukaryotic translation initiation factor 2α (eIF2α) by protein kinase RNA-like ER kinase (PERK) is a key step for adaptive mechanisms, resulting in translational inhibition. In persistent ER stress, UPR can lead to C/EBP homologous protein-induced ROS accumulation and apoptosis through the sustained phosphorylated state of eIF2α and p53-dependent pathways, for which PHLDA3 has been shown to serve as an effector.^10,25^ Although neither overexpression nor KD of PHLDA3 in control or ALS astrocytes affected cell viability, KD did reduce ROS production in ALS astrocytes characterized by proteostasis disturbances and ER stress. This finding is corroborated by a recent study demonstrating a reduction of ROS by PHLDA3-KD in cardiomyocytes exposed to oxidative stress.^9^ Thus, our results indicate that PHLDA3 can facilitate homeostatic changes in astrocytes.^24^

The human cell culture platform allowed us to explore whether *PHLDA3* expression-related changes in ALS spinal astrocytes could directly affect non-cell autonomous pathogenesis relevant to astrocyte–neuron interactions in ALS.^1^ ALS astrocytes with a greater PHLDA3 content triggered a neuronal stress response indicated by SG accumulation^22,26^ or potentiated this when induced by SA, while control astrocytes had only a limited effect. However, the reduction in *PHLDA3* expression in both control and ALS astrocytes had a protective effect against SA-induced neuronal cell stress. This also suggests that PHLDA3 levels and homeostasis in human control astrocytes were not entirely physiological, and the reduction of the PHLDA3 protein provided some neuronal protection. Notably, this beneficial effect was only observed on spinal neurons, not on cortical neurons, which support previously observed functional cell type and region specificity in astrocyte–neuron interactions and restorative processes.^27-29^ Altogether, our findings suggest that inhibiting PHLDA3 signalling could facilitate the astrocytic neurosupportive role.

A limitation of our study is that we did not directly address the precise nature of the stress-inducing or rescue mechanisms exerted by the secretome of ALS astrocytes with unaltered or lowered PHLDA3 levels, respectively. ROS is one of the few experimentally proven culprits that may underlie astrocyte-driven motor neuron death in ALS,^27,28,30^ along with recently identified potential candidates.^31^ The reduction of ROS levels detected in the PHLDA3 siRNA ACM of ALS astrocytes is one of the plausible protective mechanisms underlying limited SG accumulation.^26^ However, it is not the case for control astrocytes in which ROS production remained unchanged upon PHLDA3-KD, yet their ACM provided protection against SA-induced neuronal stress. This suggests that other PHLDA3 reduction-related cellular events are sufficient to protect against limited SA-induced neuronal SG accumulation, which was observed for control astrocytes. Since PHLDA3 also plays a role as an effector in the detrimental signalling arm of UPR,^10^ a promising but not yet successfully exploited therapeutic target in neurodegenerative diseases,^4,32^ it should be further explored how adaptive responses can lead to its activation and detrimental effects in astrocytes.

## Conclusion

In conclusion, our work supports studies showing that PHLDA3 can be differentially regulated in various cell types and states and could lead to varying outcomes. Here, we provide evidence that it does not trigger spinal cord astrocyte death, yet it can facilitate its transformation to less supportive cells for spinal neurons, with relevance to neurodegenerative pathologies.

## Supplementary Material

fcae244_Supplementary_Data

## Data Availability

Data that support the results are provided in the Supplementary Information or are available from the corresponding authors upon request. No new codes were generated in this study, and software details and availability are included in the Materials and methods.

## References

[1] Phatnani H, Maniatis T. Astrocytes in neurodegenerative disease. Cold Spring Harb Perspect Biol. 2015;7(6):1–18. 10.1101/cshperspect.a020628 PMC4448607 25877220

[2] Vahsen BF, Gray E, Thompson AG, et al Non-neuronal cells in amyotrophic lateral sclerosis—From pathogenesis to biomarkers. Nat Rev Neurol. 2021;17(6):333–348. 33927394 10.1038/s41582-021-00487-8

[3] Escartin C, Galea E, Lakatos A, et al Reactive astrocyte nomenclature, definitions, and future directions. Nat Neurosci. 2021;24:312–325. 33589835 10.1038/s41593-020-00783-4 PMC8007081

[4] Smith HL, Freeman OJ, Butcher AJ, et al Astrocyte unfolded protein response induces a specific reactivity state that causes non-cell-autonomous neuronal degeneration. Neuron. 2020;105(5):855–866.e5. 31924446 10.1016/j.neuron.2019.12.014 PMC7054837

[5] Szebényi K, Wenger LMD, Sun Y, et al Human ALS/FTD brain organoid slice cultures display distinct early astrocyte and targetable neuronal pathology. Nat Neurosci. 2021;24(11):1542–1554. 34675437 10.1038/s41593-021-00923-4 PMC8553627

[6] Hetz C, Mollereau B. Disturbance of endoplasmic reticulum proteostasis in neurodegenerative diseases. Nat Rev Neurosci. 2014;15(4):233–249. 24619348 10.1038/nrn3689

[7] Tyzack GE, Hall CE, Sibley CR, et al A neuroprotective astrocyte state is induced by neuronal signal EphB1 but fails in ALS models. Nat Commun. 2017;8(1):1164. 29079839 10.1038/s41467-017-01283-z PMC5660125

[8] Bensellam M, Chan JY, Lee K, et al Phlda3 regulates beta cell survival during stress. Sci Rep. 2019;9(1):12827. 31492921 10.1038/s41598-019-49289-5 PMC6731300

[9] Meng X, Zhang L, Han B, Zhang Z. PHLDA3 inhibition protects against myocardial ischemia/reperfusion injury by alleviating oxidative stress and inflammatory response via the Akt/Nrf2 axis. Environ Toxicol. 2021;36(11):2266–2277. 34351043 10.1002/tox.23340

[10] Han CY, Lim SW, Koo JH, Kim W, Kim SG. PHLDA3 overexpression in hepatocytes by endoplasmic reticulum stress via IRE1–Xbp1s pathway expedites liver injury. Gut. 2016;65(8):1377–1388. 25966993 10.1136/gutjnl-2014-308506 PMC4975835

[11] Qiao M, Wu M, Shi R, Hu W. PHLDA3 impedes somatic cell reprogramming by activating Akt-GSK3β pathway. Sci Rep. 2017;7(1):2832. 28588267 10.1038/s41598-017-02982-9 PMC5460190

[12] Van Harten ACM, Phatnani H, Przedborski S. Non-cell-autonomous pathogenic mechanisms in amyotrophic lateral sclerosis. Trends Neurosci. 2021;44(8):658–668. 34006386 10.1016/j.tins.2021.04.008 PMC8972039

[13] Noble M, Murray K. Purified astrocytes promote the in vitro division of a bipotential glial progenitor cell. EMBO J. 1984;3(10):2243–2247. 6542000 10.1002/j.1460-2075.1984.tb02122.x PMC557676

[14] Tyzack GE, Sitnikov S, Barson D, et al Astrocyte response to motor neuron injury promotes structural synaptic plasticity via STAT3-regulated TSP-1 expression. Nat Commun. 2014;5:4294. 25014177 10.1038/ncomms5294 PMC4104454

[15] Hall CE, Yao Z, Choi M, et al Progressive motor neuron pathology and the role of astrocytes in a human stem cell model of VCP-related ALS. Cell Rep. 2017;19(9):1739–1749. 28564594 10.1016/j.celrep.2017.05.024 PMC5464993

[16] Fernandopulle MS, Prestil R, Grunseich C, Wang C, Gan L, Ward ME. Transcription factor–mediated differentiation of human iPSCs into neurons. Curr Protoc Cell Biol. 2018;79(1):e51. 29924488 10.1002/cpcb.51 PMC6993937

[17] Chen H, Qian K, Du Z, et al Modeling ALS with iPSCs reveals that mutant SOD1 misregulates neurofilament balance in motor neurons. Cell Stem Cell. 2014;14(6):796–809. 24704493 10.1016/j.stem.2014.02.004 PMC4230530

[18] Schindelin J, Arganda-Carreras I, Frise E, et al Fiji: An open-source platform for biological-image analysis. Nat Methods. 2012;9(7):676–682. 22743772 10.1038/nmeth.2019 PMC3855844

[19] Carpenter AE, Jones TR, Lamprecht MR, et al CellProfiler: Image analysis software for identifying and quantifying cell phenotypes. Genome Biol. 2006;7(10):R100. 17076895 10.1186/gb-2006-7-10-r100 PMC1794559

[20] Liu J, Liu X, Hui X, et al Novel role for pleckstrin homology-like domain family A, member 3 in the regulation of pathological cardiac hypertrophy. J Am Heart Assoc. 2019;8(16):e011830. 31426686 10.1161/JAHA.118.011830 PMC6759890

[21] Lei L, Wang Y, Li ZH, et al PHLDA3 promotes lung adenocarcinoma cell proliferation and invasion via activation of the Wnt signaling pathway. Lab Investig. 2021;101(9):1130–1141. 34006890 10.1038/s41374-021-00608-3

[22] Sidibé H, Dubinski A, Vande Velde C. The multi-functional RNA-binding protein G3BP1 and its potential implication in neurodegenerative disease. J Neurochem. 2021;157(4):944–962. 33349931 10.1111/jnc.15280 PMC8248322

[23] Kawase T, Ohki R, Shibata T, et al PH Domain-Only protein PHLDA3 is a p53-regulated repressor of Akt. Cell. 2009;136(3):535–550. 19203586 10.1016/j.cell.2008.12.002

[24] Hetz C, Saxena S. ER stress and the unfolded protein response in neurodegeneration. Nat Rev Neurol. 2017;13(8):477–491. 28731040 10.1038/nrneurol.2017.99

[25] Maher H, Zeeshan A, Lee GH, Kim H-R, Chae H-J. Molecular sciences endoplasmic reticulum stress and associated ROS. Int J Mol Sci. 2016;17(3):327. 26950115 10.3390/ijms17030327 PMC4813189

[26] Somasekharan SP, Zhang F, Saxena N, et al G3BP1-linked mRNA partitioning supports selective protein synthesis in response to oxidative stress. Nucleic Acids Res. 2020;48(12):6855–6873. 32406909 10.1093/nar/gkaa376 PMC7337521

[27] Di Giorgio FP, Carrasco MA, Siao MC, Maniatis T, Eggan K. Non–cell autonomous effect of glia on motor neurons in an embryonic stem cell–based ALS model. Nat Neurosci. 2007;10(5):608–614. 17435754 10.1038/nn1885 PMC3139463

[28] Nagai M, Re DB, Nagata T, et al Astrocytes expressing ALS-linked mutated SOD1 release factors selectively toxic to motor neurons. Nat Neurosci. 2007;10(5):615–622. 17435755 10.1038/nn1876 PMC3799799

[29] Tsai H-H, Li H, Fuentealba LC, et al Regional astrocyte allocation regulates CNS synaptogenesis and repair. Science. 2012;337:358–362. 22745251 10.1126/science.1222381 PMC4059181

[30] Fritz E, Izaurieta P, Weiss A, et al Mutant SOD1-expressing astrocytes release toxic factors that trigger motoneuron death by inducing hyperexcitability. J Neurophysiol. 2013;109(11):2803–2814. 23486205 10.1152/jn.00500.2012 PMC3680799

[31] Mishra V, Re DB, Le Verche V, et al Systematic elucidation of neuron-astrocyte interaction in models of amyotrophic lateral sclerosis using multi-modal integrated bioinformatics workflow. Nat Commun. 2020;11(1):5579. 33149111 10.1038/s41467-020-19177-y PMC7642391

[32] Das I, Krzyzosiak A, Schneider K, et al Preventing proteostasis diseases by selective inhibition of a phosphatase regulatory subunit. Science. 2015;348:239–242. 25859045 10.1126/science.aaa4484 PMC4490275

