# Supplementary material for: Inhibition of PHLDA3 expression in human superoxide dismutase 1-mutant amyotrophic lateral sclerosis astrocytes protects against neurotoxicity
Source: Brain Commun. 2024 Jul 25;6(4):fcae244. doi: 10.1093/braincomms/fcae244 (PMC11323778; doi:10.1093/braincomms/fcae244)
Supplement: fcae244_Supplementary_Data [file fcae244_supplementary_data.pdf]

Supplementary Figures

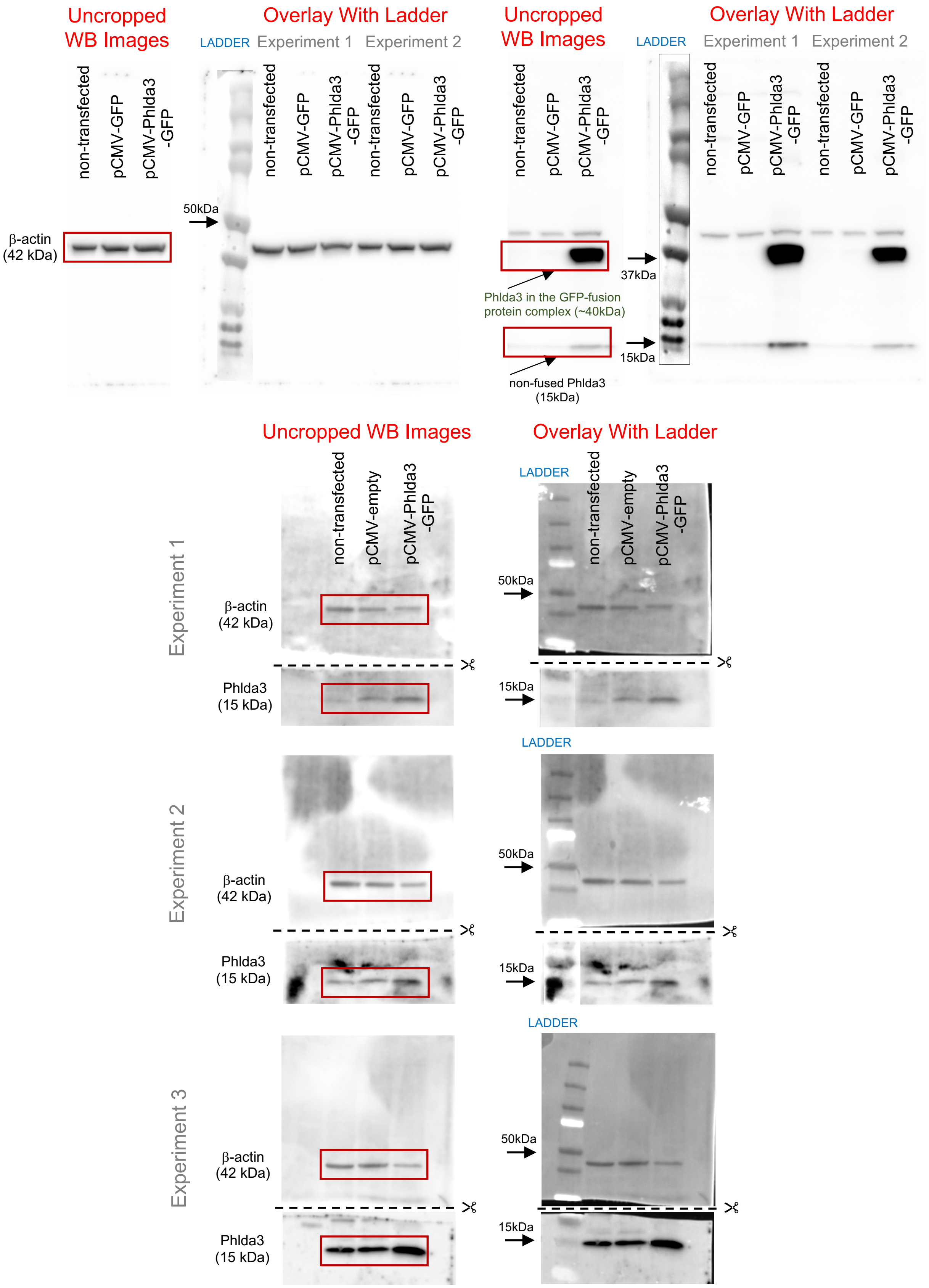

Supplementary Fig 1 Uncropped western blot (WB) images (left; luminescence channel) and their overlays with the corresponding images of molecular weight markers (right; luminescence channel or light channel) for Fig. 2B. Sample labels refer to non-transfected or transfected mouse astrocyte cultures. Dashed lines indicate blot membrane cuts that enabled simultaneous detection of multiple proteins in the same sample.

Uncropped WB Images
Overlayed With Ladder

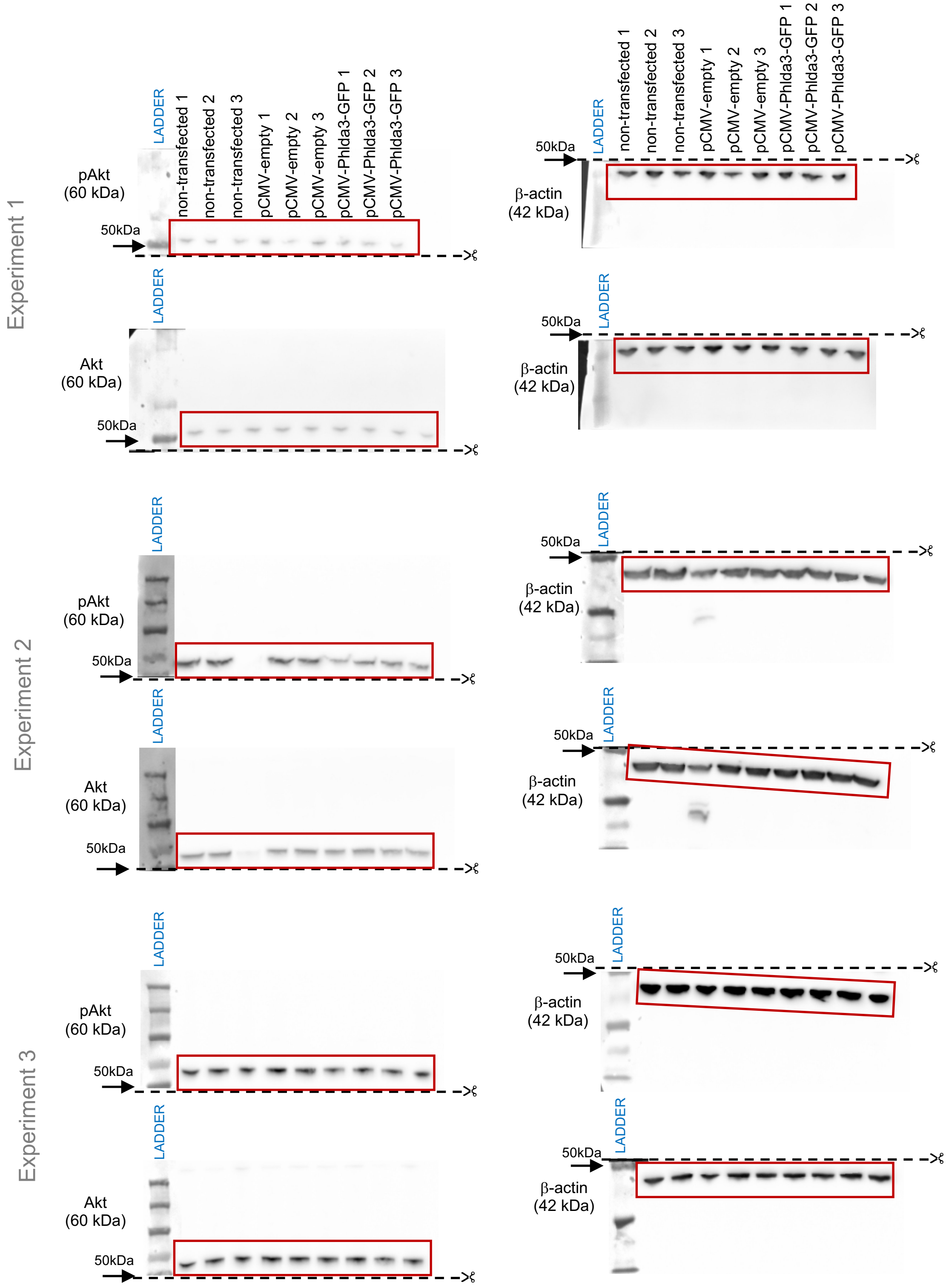

Supplementary Fig 2 Uncropped western blot (WB) images (luminescence channel) overlayed with the corresponding images of molecular weight markers (luminescence channel or light channel) for Fig. 2D. Sample labels refer to non-transfected or transfected mouse astrocyte cultures. Dashed lines indicate blot membrane cuts that enabled simultaneous detection of multiple proteins in the same sample.

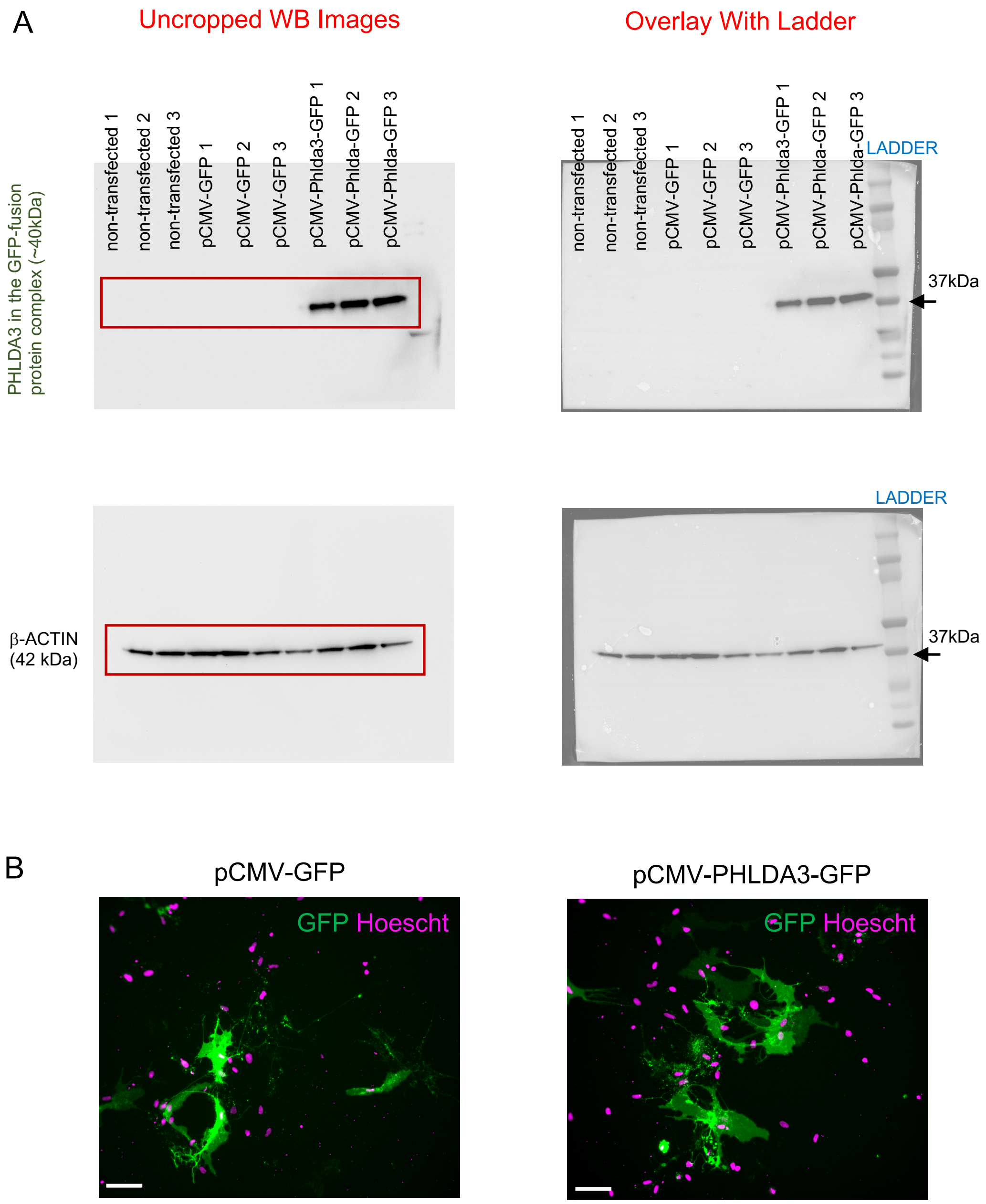

Supplementary Fig 3 A. Uncropped western blot (WB) images (left; luminescence channel) and their overlays with the corresponding images of molecular weight markers (right; light channel) for Fig. 3B. Sample labels refer to non-transfected or transfected human control astrocyte cultures. **B.** Representative merged immunofluorescence images of human control astrocytes transfected by pCMV-GFP (left) or pCMV-PHLDA3-GFP (right), showing GFP immunofluorescence and Hoescht nuclear staining. Scale bar: 35 μm.

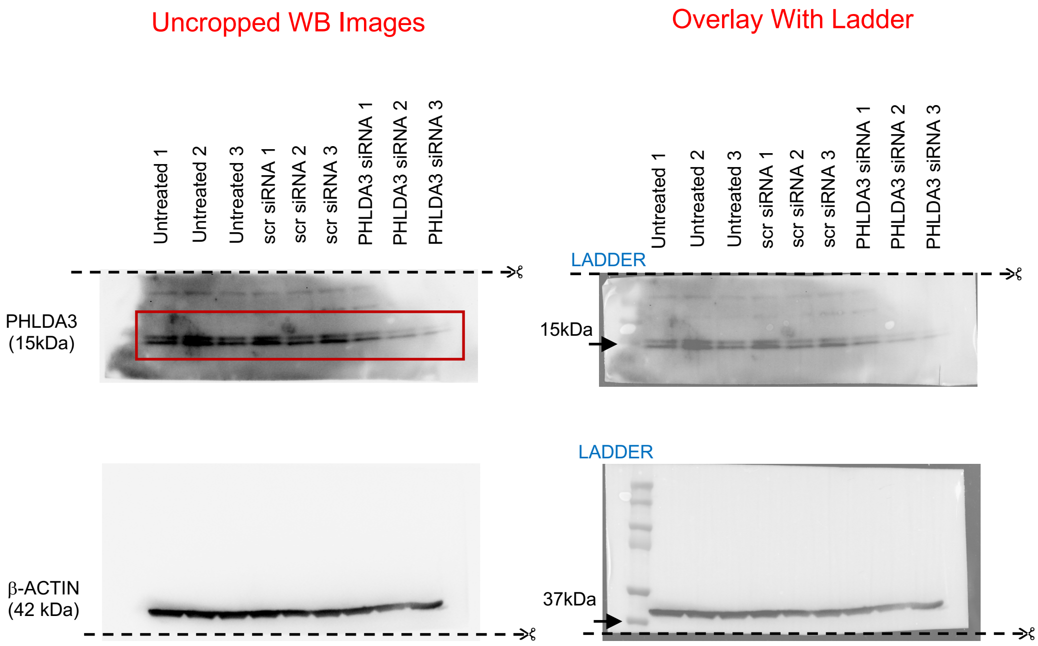

Supplementary Fig 4 Uncropped western blot (WB) images (left; luminescence channel) and their overlays with the corresponding images of molecular weight markers (right; light channel) for Fig. 3D. Sample labels refer to non-transfected or transfected human control astrocyte cultures. Dashed lines indicate blot membrane cuts that enabled simultaneous detection of multiple proteins in the same sample.

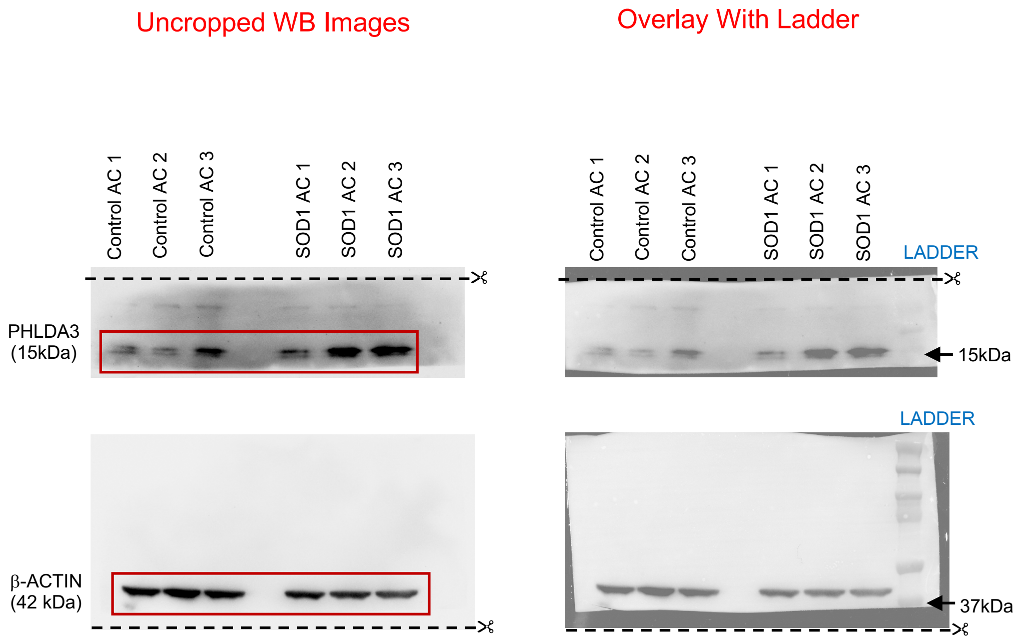

Supplementary Fig 5 Uncropped western blot (WB) images (left; luminescence channel) and their overlays with the corresponding images of molecular weight markers (right; light channel) for Fig. 4B. Sample labels refer to human control or SOD1 ALS astrocyte cultures. Dashed lines indicate blot membrane cuts that enabled simultaneous detection of multiple proteins in the same sample.

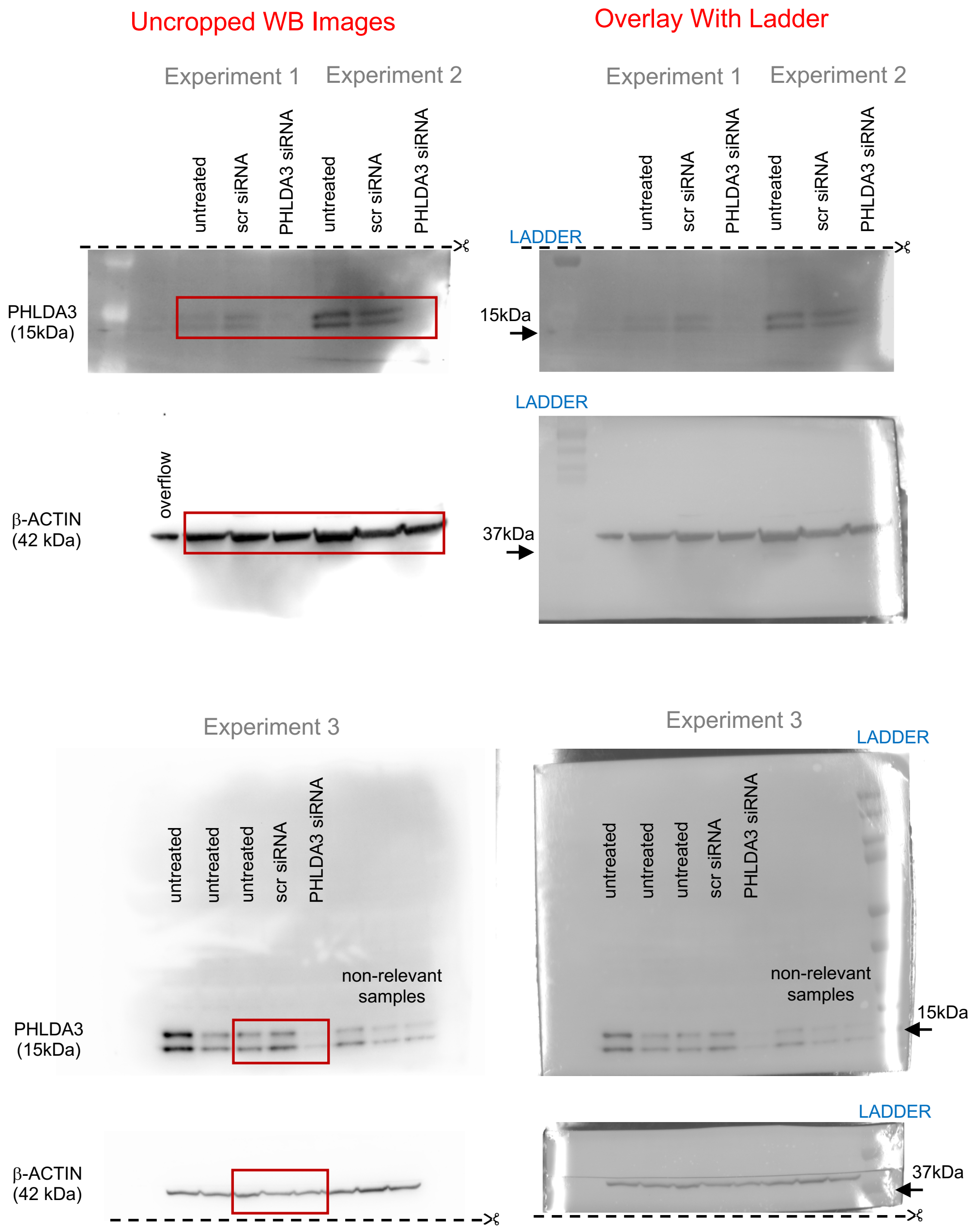

Supplementary Fig 6 Uncropped western blot (WB) images (left; luminescence channel) and their overlays with the corresponding images of molecular weight markers (right; light channel) for Fig. 4D. Sample labels refer to non-transfected or transfected human SOD1 ALS astrocyte cultures. Dashed lines indicate blot membrane cuts that enabled simultaneous detection of multiple proteins in the same sample.

Supplementary Table 1

primary Ab	source	dilution	species	RRID reference
**β-actin**	Sigma A2228	1:1000 for WB	Mouse	RRID:AB_476697
**ACTB-HRP**	Proteintech HRP-60008	1:10000 for WB	Mouse	RRID:AB_2819183
**Akt**	Cell Signaling 9272	1:1000 for WB	Rabbit	RRID:AB_329827
**pAkt**	Cell Signaling 4060	1:2000 for WB	Rabbit	RRID:AB_2315049
**CHAT**	Sigma Aldrich AB144P	1:200 for ICC	Goat	RRID:AB_2079751
**G3BP1**	Proteintech 13057-2-AP	1:500 for ICC	Rabbit	RRID:AB_2232034
**GFAP**	Antibodies.com A85307	1:800 for ICC	Chicken	RRID:AB_2748894
**GFAP**	Abcam ab4674	1:500 for ICC	Chicken	RRID:AB_304558
**GFP**	ThermoFisher A10262	1:1000 for ICC/WB	Chicken	RRID:AB_2534023
**NeuN**	Abcam ab104224	1:200 for ICC	Mouse	RRID:AB_10711040
**Phlda3**	Novus Biologicals NBP1-56772	1:200 for WB	Rabbit	RRID:AB_11035294
**Phlda3**	LSBio LS-C385473	1:150 for ICC<br>1:500 for WB	Rabbit	https://www.lsbio.com/antibodies/phlda3-antibody-40-120-aa-internal-elisa-ihc-wb-western-ls-c385473/397574#validation-section

secondary Ab	source	dilution	species	RRID reference
**Anti-Rabbit IgG-HRP**	ThermoFisher 31462	1:5000 for WB	Goat	RRID:AB_228338
**Anti-Rabbit IgG-HRP**	Jackson ImmunoResearch 111-035-003	1:5000 for WB	Goat	RRID:AB_2313567
**Anti-Mouse IgG-HRP**	Proteintech SA00001-1	1:5000 for WB	Goat	RRID:AB_2722565
**Anti-Mouse IgG-HRP**	Jackson ImmunoResearch 115-035-003	1:5000 for WB	Goat	RRID:AB_10015289
**Anti-Rabbit Alexa 488**	ThermoFisher A-11008	1:500 for ICC	Goat	RRID:AB_143165
**Anti-Chicken Alexa Fluor 488**	ThermoFisher A-11039	1:200 for ICC	Goat	RRID:AB_2534096
**Anti-Rabbit Alexa Fluor 546**	ThermoFisher A-11035	1:200 for ICC	Goat	RRID:AB_2534093
**Anti-Chicken Alexa Fluor-647**	Abcam ab150175	1:500 for ICC	Goat	RRID:AB_2732800
**Anti-Goat IgG 488**	ThermoFisher SA5-10086	1:500 for ICC	Donkey	RRID:AB_2556666

Supplementary Table 1 List of antibodies used.

Supplementary Table 2

figure number	statistical tests	n-values, sample sizes	data representation	p-value	further details
**Fig 1A**	no statistical test	N=3 cultures for each condition	mean±SD	N/A	demonstration of distribution
**Fig 1B**	unpaired two-tailed t-test	N=5 independently transfected cultures	mean±SEM	0.6105	t=0.530, df=8<br>F, DFn, Dfd<br>2.58, 4, 4
**Fig 2B**	unpaired two-tailed t-test	N=3 independently transfected cultures	mean±SEM	0.0497	t=2.78, df=4<br>F, DFn, Dfd<br>2.04, 2, 2
**Fig 2C**	two-tailed one-way ANOVA	N=4 independently transfected cultures	mean±SEM	0.8581	F (2, 9) = 0.1557
**Fig 2D**	unpaired two-tailed t-test	N=3 independently transfected cultures	mean±SEM	0.2120	t=1.48, df=4
**Fig. 3C (left)**	two-tailed one-way ANOVA	N=3 independently transfected cultures	mean±SEM	ANOVA 0.8660	F (2, 6) = 0.1473
**Fig. 3C (right)**	two-tailed one-way ANOVA	N=6 independently transfected cultures	mean±SEM	ANOVA 0.8534	F (2, 15) = 0.1602
**Fig. 3D**	two-tailed one-way ANOVA	N=3 independently transfected cultures	mean±SEM	ANOVA 0.0245	F (2, 6) = 7.328
**Fig. 3E (left)**	two-tailed one-way ANOVA	N=4 independently transfected cultures	mean±SEM	ANOVA 0.6562	F (2, 9) = 0.4416
**Fig. 3E (right)**	two-tailed one-way ANOVA Tukey's posthoc test	N=4 independently transfected cultures	mean±SEM	ANOVA 0.0146<br>Non-transf. vs. SCR RNA = 0.0358<br>Non-transf. vs. PHLDA3 KD = 0.0184<br>SCR RNA vs. PHLDA3 KD = 0.9043	F (2, 9) = 7.008
**Fig 4A**	unpaired two-tailed t-test	N=6 independent cultures	mean±SEM	0.0002	t=5.75, df=10<br>8.23, 5, 5
**Fig 4B**	unpaired two-tailed t-test	N=3 independent cultures	mean±SEM	0.0418	t=2.955, df=4
**Fig 4C (left)**	unpaired two-tailed t-test	N=4 independent cultures	mean±SEM	0.2597	t=1.244, df=6
**Fig 4C (right)**	unpaired two-tailed t-test	N=4 independent cultures	mean±SEM	0.0009	t=6.030, df=6
**Fig 4D**	two-tailed one-way ANOVA Tukey's posthoc test	N=3 independently transfected cultures	mean±SEM	ANOVA 0.0087<br>Non-treated vs PHLDA3 KD = 0.0138<br>Non-treated vs SCR RNA = 0.9997<br>SCR RNA vs PHLDA3 KD = 0.0141	F (2, 6) = 11.57
**Fig 4E (left)**	two-tailed one-way ANOVA Dunnett's posthoc test	N=5 independently transfected cultures (3 wells per culture)	mean±SEM	ANOVA 0.0498<br>Non-treated vs PHLDA3 KD = 0.0315<br>Non-treated vs SCR RNA = 0.5092	F (2, 12) = 3.891
**Fig 4E (right)**	two-tailed one-way ANOVA	N=5 independently transfected cultures (3 wells per culture)	mean±SEM	ANOVA 0.3787	F (2, 12) = 1.054

**Fig 5C**	two-tailed one-way ANOVA	N=4 independently treated cultures	mean±SEM	ANOVA 0.2743	F (3, 12) = 1.462
**Fig 5D**	two-tailed one-way ANOVA, Tukey's posthoc test	N=4 independently treated cultures	mean±SEM	ANOVA 0.0047<br>SCR RNA vs. SCR RNA+SA = 0.0033<br>SCR RNA vs. PHDLA3 KD = 0.5383<br>SCR RNA vs. PHDLA3 KD+SA = 0.1278<br>SCR RNA+SA vs. PHDLA3 KD =0.0353<br>SCR RNA+SA vs. PHDLA3 KD+SA = 0.1980<br>PHDLA3 KD vs. PHDLA3 KD+SA = 0.7314	F (3, 12) = 7.334
**Fig 6C (left)**	unpaired two-tailed t-test	N=3 independent cultures	mean±SEM	0.0305	t=3.28, df=4
**Fig 6C (right)**	unpaired two-tailed t-test	N=3 independent cultures	mean±SEM	0.5897	t=0.585, df=4
**Fig 7B (left)**	two-tailed one-way ANOVA	N=3 independently treated cultures	mean±SEM	ANOVA 0.5853	F (3, 8) = 0.6861
**Fig 7B (right)**	two-tailed one-way ANOVA	N=3 independently treated cultures	mean±SEM	ANOVA 0.0681	F (3, 8) = 3.535
**Fig 7C (left)**	two-tailed one-way ANOVA, Tukey's posthoc test	N=3 independently treated cultures	mean±SEM	ANOVA 0.0007<br>SCR RNA vs. SCR RNA+SA = 0.0020<br>SCR RNA vs. PHDLA3 KD = 0.9001<br>SCR RNA vs. PHDLA3 KD+SA = 0.9812<br>SCR RNA+SA vs. PHDLA3 KD = 0.0010<br>SCR RNA+SA vs. PHDLA3 KD+SA = 0.0031<br>PHDLA3 KD vs. PHDLA3 KD+SA = 0.7232	F (3, 8) = 17.28
**Fig 7C (right)**	two-tailed one-way ANOVA, Tukey's posthoc test	N=3 independently treated cultures	mean±SEM	ANOVA 0.0051<br>SCR RNA vs. SCR RNA+SA = 0.0041<br>SCR RNA vs. PHDLA3 KD = 0.6422<br>SCR RNA vs. PHDLA3 KD+SA = 0.3240<br>SCR RNA+SA vs. PHDLA3 KD = 0.0189<br>SCR RNA+SA vs. PHDLA3 KD+SA = 0.0454<br>PHDLA3 KD vs. PHDLA3 KD+SA = 0.9175	F (3, 8) = 9.548

Supplementary Table 2 Samples sizes and statistical tests used in this work.
